# Effects of *KRAS*, *STK11*, *KEAP1*, and *TP53* mutations on the clinical outcomes of immune checkpoint inhibitors among patients with lung adenocarcinoma

**DOI:** 10.1371/journal.pone.0307580

**Published:** 2024-07-22

**Authors:** Yao Liang, Osamu Maeda, Chiaki Kondo, Kazuki Nishida, Yuichi Ando

**Affiliations:** 1 Department of Clinical Oncology and Chemotherapy, Nagoya University Hospital, Nagoya, Aichi, Japan; 2 Department of Advanced Medicine, Nagoya University Hospital, Nagoya, Aichi, Japan; CNR, ITALY

## Abstract

**Background:**

This study aimed to identify the associations between individual *KRAS*, *STK11*, *KEAP1*, or *TP53* mutations, as well as the comutation status of these genes, and the tumor mutation burden (TMB) with clinical outcomes of lung adenocarcinoma patients treated with immune checkpoint inhibitors (ICIs).

**Methods:**

We collected data from patients with lung adenocarcinoma treated with ICIs from the Center for Cancer Genomics and Advanced Therapeutics (C-CAT) database between June 2019 and August 2023. The main endpoints were the treatment response and overall survival (OS).

**Results:**

Among 343 patients with lung adenocarcinoma, 61 (18%), 69 (20%), 41 (12%), and 222 (65%) patients had *KRAS*, *STK11*, *KEAP1*, and *TP53* mutations, respectively. An overall objective response was observed in 94 of 338 patients (28%), including 2 (1%) who achieved a complete response and 92 (27%) who achieved a partial response. Patients with *STK11*, *KEAP1*, or *TP53* mutations had a significantly greater TMB (P<0.001). According to the univariate analysis, the treatment response was significantly correlated with *TP53* mutation in both the general (P = 0.041) and *KRAS* wild-type (P = 0.009) populations. *KEAP1* and *TP53* mutations were associated with worse OS among assessable patients (hazard ratio (HR) = 2.027, P = 0.002; HR = 1.673, P = 0.007, respectively) and among patients without *KRAS* mutations (HR = 1.897, P = 0.012; HR = 1.908, P = 0.004, respectively). According to the multivariate analysis, *KEAP1* (HR = 1.890, P = 0.008) and *TP53* (HR = 1.735, P = 0.011) mutations were found to be independent factors for OS.

**Conclusions:**

*STK11*, *KEAP1*, and *TP53* mutations are significantly associated with a high TMB. *TP53* mutation could affect the treatment response to some degree, and both *KEAP1* and *TP53* mutations resulted in inferior OS in the general patient population and in those with *KRAS-*wild-type lung adenocarcinoma, indicating that *KEAP1* and *TP53* mutations might act as prognostic factors for ICI treatment in lung adenocarcinoma patients.

## Introduction

Over the last decade, the treatment for non-small cell lung cancer (NSCLC) has improved by the use of immune checkpoint inhibitors (ICIs), especially inhibitors targeting programmed death-1 (PD-1) or programmed death-ligand 1 (PD-L1) [1]. Although ICIs have provided favorable clinical outcomes, such as improved progression-free survival (PFS) and overall survival (OS), an objective response (OR) was not observed in most patients with NSCLC [2, 3]. Although PD-L1 and the tumor mutation burden (TMB) are known to be predictive biomarkers of the response to ICIs, given that their predictive ability is limited in clinical practice [4], other biomarkers of the response to ICIs and the association between those biomarkers and the TMB must be explored to optimize treatment for patients with NSCLC.

Alterations in several tumor genes, such as mutations in *KRAS*, *STK11*, and *KEAP1*, have been suggested as potential biomarkers for the ICI response in patients with NSCLC [5]. *KRAS* mutations are the most common clonal oncogenic driver in NSCLC and are present in 35% of lung adenocarcinomas [6]. *STK11* encodes the tumor suppressor liver kinase B1 (LKB1), which suppresses tumor growth, and its mutation occurs in approximately 30% of *KRAS*-mutant lung adenocarcinoma cases; this mutation can promote *KRAS*-driven cancer growth and early metastasis [7, 8]. *KEAP1* encodes Kelch-like ECH-associated protein 1 (KEAP1), which negatively regulates nuclear factor erythroid 2-related factor 2 (NRF2), a regulator of cell survival. Loss-of-function mutations in *KEAP1*, which account for approximately 20% of *KRAS*-mutant NSCLC cases, lead to NRF2 activation, resulting in accelerated tumor growth and chemoresistance [7, 9]. *TP53* encodes the p53 tumor suppressor protein, a master regulator of the cell cycle and cell death [10]. *TP53* mutations were found in approximately 42% of patients with *KRAS*-mutant NSCLC, and comutation with *KRAS* was linked to an inflammatory tumor microenvironment [5, 11]. Moreover, *KRAS* mutation can upregulate NRF2 signal transduction, contributing to oncogenic transformation and senescence evasion [12]. Compared to individual *STK11*, *KEAP1*, or *TP53* mutations, comutation of *KRAS* and *STK11*, *KEAP1*, or *TP53* was associated with worse or better clinical responses to ICIs in patients with NSCLC [7, 8, 13]. However, only a few studies have explored the impact of *STK11*, *KEAP1*, and *TP53* mutations on clinical outcomes among populations with wild-type *KRAS*. In this study, we aimed to identify the associations between individual mutations in *KRAS*, *STK11*, *KEAP1*, or *TP53*, as well as the comutation status of these genes, and the TMB with clinical outcomes of patients with lung adenocarcinoma treated with immune checkpoint inhibitors (ICIs).

## Materials and methods

### Patients

We collected data from patients with lung cancer who had undergone ICI therapy and who had received a cancer genomic medicine test, including OncoGuide™ NCC Oncopanel, FoundationOne^®^ CDx and FoundationOne^®^ Liquid CDx, who were enrolled in the Center for Cancer Genomics and Advanced Therapeutics (C-CAT) database between June 1, 2019, and August 30, 2023. All authors had access to information that could identify individual participants during or after data collection. This study was conducted in accordance with the Ethical Guidelines for Medical and Biological Research Involving Human Subjects (Ministry of Health, Labor and Welfare, Japan) and the Declaration of Helsinki. This study was approved by the Institutional Review Board of Nagoya University Hospital (approval no. 2022–0025) and by the review board of C-CAT (C-CAT Control Number: CDU2022-030N). Written consent was obtained from all participants before study initiation.

### Data analysis

The TMB was defined as the total number of mutations per coding area of a tumor genome [14]. TMB-high was defined as ≥10 mutations/Mb, and TMB-low was defined as <10 mutations/Mb for OncoGuide™ NCC Oncopanel and FoundationOne^®^ CDx, while the cutoff value was 16 mutations/Mb for FoundationOne^®^ Liquid CDx [15–17]. PD-L1 expression was tested using the Dako PD-L1 immunohistochemistry (IHC) 22C3 pharmDx assay for pembrolizumab, the Ventana OptiView PD-L1 (SP142) assay for atezolizumab, and the Ventana OptiView PD-L1 (SP263) assay for adjuvant atezolizumab. PD-L1 positivity was defined as ≥1% of tumor cells staining positive for PD-L1. The main endpoints were the best objective response during ICI treatment and OS. The objective response rate was evaluated according to the Response Evaluation Criteria in Solid Tumors (RECIST) version 1.1 [18]. OS was defined as the time from the date of receiving ICI to the date of death from any cause or the last confirmation of survival. In this study, age, sex, Eastern Cooperative Oncology Group performance status (ECOG PS), smoking history, PD-L1 positivity status, TMB status, treatment line, and regimen were considered potential clinical factors associated with the treatment response and OS. The associations of these clinical variables and target genes, including *KRAS*, *STK11*, *KEAP1*, *TP53*, and other driver genes, with the treatment response and OS were assessed.

### Statistical analysis

The influences of the mutation status of a single gene, including *KRAS*, *STK11*, *KEAP1*, and *TP53*, on the TMB were compared with the Mann‒Whitney U test, while the effects of the *STK11*, *KEAP1*, and *TP53* mutation statuses according to *KRAS* on the TMB were analyzed with Friedman’s test followed by the Bonferroni correction. Univariate analyses with two-sided Fisher’s exact tests were conducted to evaluate the impacts of *KRAS*, *STK11*, *KEAP1*, and *TP53* mutation statuses on the PD-L1 status and treatment response. Multivariate analysis was performed to test the associations between clinical and genetic variables and the treatment response, and the results are shown as odds ratios (ORs) with 95% confidence intervals (CIs). The Kaplan‒Meier method was used to analyze and compare OS between groups with differences in the mutation status of the four genes. Hazard ratios (HRs) in univariate and multivariate analyses of OS, with the corresponding 95% CIs, were calculated using the Cox proportional hazard model. All clinical and genetic variables in the multivariate models were selected using a stepwise method. Given the exploratory nature of this study, which aimed to identify potential associations, P values were not adjusted for multiple comparisons to avoid missing potential signals. All reported P values less than 0.05 were considered to indicate statistical significance. All the statistical analyses were performed using IBM SPSS Statistics version 29.0 (IBM Japan Ltd., Tokyo, Japan).

## Results

A total of 509 patients with lung cancer received ICIs, 343 of whom had lung adenocarcinoma (Table 1). *KRAS*, *STK11*, *KEAP1*, and *TP53* mutations were detected in 62 (18%), 70 (20%), 42 (12%), and 222 (65%) patients with lung adenocarcinoma, respectively. A total of 77 (22%), 71 (21%), 71 (21%), 39 (11%), and 36 (10%) patients had alterations in other driver genes, including *EGFR*, *HER2*, *NTRK*, *MET*, and *ROS1*, respectively. A TMB-high status and PD-L1 positivity were present in 103 (30%) and 160 (47%) patients, respectively. The overall best objective response was found in 96 of 343 patients (28%), including 2 (1%) who achieved a complete response (CR) and 94 (27%) who achieved a partial response (PR).

**Table 1:** Patient characteristics, n (%).

Characteristics	No. of patients (n = 343)
Age (years), median (range)	64 (20–87)
Sex	
Male	199 (58)
Female	144 (42)
Smoking status	
Current/Former	213 (62)
Never	123 (36)
Unknown	7 (2)
ECOG performance status	
0–1	312 (91)
≥2	18 (5)
Unknown	13 (4)
Histopathology	
Adenocarcinoma	343 (100)
Gene mutation	
*STK11*	70 (20)
*KEAP1*	42 (12)
*TP53*	222 (65)
*KRAS+STK11*	14 (4)
*KRAS+KEAP1*	6 (2)
*KRAS+TP53*	27 (8)
*STK11+KEAP1+TP53*	9 (3)
*KRAS+STK11+KEAP1+TP53*	0
Alterations in other driver genes	
*EGFR*	77 (22)
*HER2*	71 (21)
*NTRK*	71 (21)
*MET*	39 (11)
*ROS1*	36 (10)
*BRAF*	30 (9)
*ALK*	20 (6)
*RET*	18 (5)
Therapy line for ICIs	
1^st^	127 (37)
≥2^nd^	215 (63)
Unknown	1 (0)
Regimen type	
Anti-PD-(L)1 antibody alone	202 (59)
Anti-PD-1 and anti-CTLA-4 antibodies	4 (1)
Anti-PD-(L)1 antibody and chemotherapy	113 (33)
Anti-PD-(L)1 and anti-CTLA-4 antibodies and chemotherapy	4 (1)
Anti-PD-(L)1 and anti-VEGF antibodies and chemotherapy	20 (6)
TMB status	
TMB-high	103 (30)
TMB-low	240 (70)
PD-L1 status	
Positive	160 (47)
Negative	136 (40)
Unknown	47 (14)
Objective response	
Complete response	2 (1)
Partial response	94 (27)
Stable disease	113 (33)
Progressive disease	104 (30)
Unknown	30 (9)
Genomic medicine test	
OncoGuide™ NCC Oncopanel	71 (21)
FoundationOne^®^ CDx	261 (76)
FoundationOne^®^ Liquid CDx	11 (3)

Abbreviation: ECOG, Eastern Cooperative Oncology Group; ICIs, immune checkpoint inhibitors; TMB, tumor mutation burden; PD-(L)1, programmed death-(ligand) 1; CTLA-4, cytotoxic T-lymphocyte antigen 4; VEGF; vascular endothelial growth factor.

**Fig 1:**
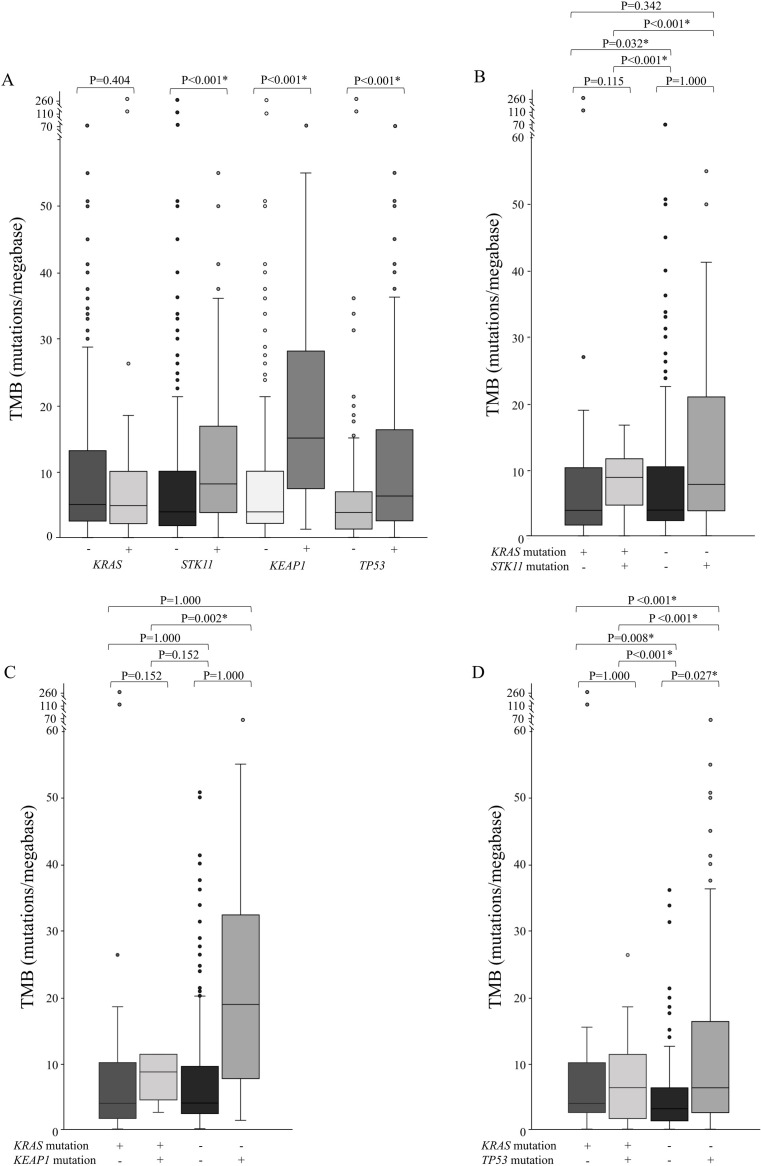
TMB according to *KRAS*, *STK11*, *KEAP1*, and *TP53* statuses. (A) TMB according to the single-gene status of *KRAS*, *STK11*, and *KEAP1*. (B) TMB according to the comutation status of *KRAS* and *STK11*. (C) TMB according to the comutation status of *KRAS* and *KEAP1*. (D) TMB according to the comutation status of *KRAS* and *TP53*. Note: * P<0.05 was considered to indicate statistical significance. Abbreviations: TMB, tumor mutation burden; WT, wild-type; MUT, mutant-type.

Among the 296 patients whose PD-L1 expression status was recorded, we explored the associations between the *KRAS*, *STK11*, *KEAP1*, and *TP53* statuses and the PD-L1 expression status (S1 Table). A tendency for patients with *STK11* mutant-type (OR, 0.664, 95% CI: 0.380–1.161, P = 0.157) and *STK11* mutant but *KRAS* wild-type (OR, 0.614, 95% CI: 0.326–1.157, P = 0.150) phenotypes to be less likely to exhibit PD-L1 positivity was observed, whereas patients with *TP53* mutant-type (OR, 1.583, 95% CI: 0.976–2.568, P = 0.066) and those with comutations of *KRAS* (OR, 4.857, 95% CI: 1.450–16.266, P = 0.013) tended to exhibit PD-L1 positivity. We also investigated the effects of the *KRAS*, *STK11*, *KEAP1*, and *TP53* statuses on the TMB among all patients with lung adenocarcinoma (Fig 1). Patients with *STK11*, *KEAP1*, or *TP53* mutations had a significantly greater TMB (both P<0.001). When the TMB distribution was evaluated according to the *KRAS* status, we found that patients carrying *TP53* mutations had a significantly greater TMB in the *KRAS*-wild-type population than in the other three subtypes (all P<0.05, Fig 1). In addition, an increased TMB was observed among patients with single mutations in *STK11* (P<0.001), *KEAP1* (P = 0.002), or *TP53* (P<0.001) compared with patients with comutation of *KRAS*.

Through the univariate analysis, we evaluated whether the *KRAS*, *STK11*, *KEAP1*, and *TP53* statuses correlated with the treatment response among the 313 patients with known data and found that mutant *TP53* (OR, 1.767, 95% CI: 1.045–2.991, P = 0.041) and mutant *TP53* but wild-type *KRAS* (OR, 2,321, 95% CI: 1.240–4.347, P = 0.009) were significantly associated with the objective response (Table 2). Multivariate analysis was performed to explore the relationships between covariables, including age, sex, ECOG PS, smoking history, PD-L1 positivity, TMB status, treatment line, regimen, mutation statuses of *KRAS*, *STK11*, *KEAP1*, and *TP53*, and treatment response. Patients who were aged 65 years or older (OR, 1.945, 95% CI: 1.089–3.474, P = 0.025), who were PD-L1-positive (OR, 2.444, 95% CI: 1.354–4.409, P = 0.003), who had a high TMB (OR, 2.706, 95% CI: 1.478–4.954, P = 0.001), who received ICIs as a first-line therapy (OR, 2.093, 95% CI: 1.077–4.065, P = 0.029) or who received ICIs in combination with chemotherapy (OR, 0.409, 95% CI: 0.205–0.814, P = 0.011) tended to achieve an objective response (Table 3). No significant association existed between mutations in the four genes and the treatment response.

**Table 2:** Univariate analysis of the treatment response according to the *KRAS*, *STK11*, *KEAP1*, and *TP53* status.

Gene status	No. of patients without an objective response	No. of patients with an objective response	OR	95% CI	P
*KRAS* ^MUT^	40	16	0.885	0.468–1.673	0.752
*KRAS* ^WT^	177	80			
*STK11* ^MUT^	37	20	1.280	0.698–2.348	0.431
*STK11* ^WT^	180	76			
*KEAP1* ^MUT^	24	15	1.489	0.743–2.985	0.269
*KEAP1* ^WT^	193	81			
*TP53* ^MUT^	131	70	1.767	1.045–2.991	0.041*
*TP53* ^WT^	86	26			
*KRAS*^MUT^/*STK11*^MUT^	8	2	0.571	0.107–3.041	0.707
*KRAS*^MUT^/*STK11*^WT^	32	14			
*KRAS*^WT^/*STK11*^MUT^	29	18	1.482	0.767–2.863	0.295
*KRAS*^WT^/*STK11*^WT^	148	62			
*KRAS*^MUT^/*KEAP1*^MUT^	4	2	1.286	0.211–7.826	1.000
*KRAS*^MUT^/*KEAP1*^WT^	36	14			
*KRAS*^WT^/*KEAP1*^MUT^	20	13	1.523	0.716–3.239	0.315
*KRAS*^WT^/*KEAP1*^WT^	157	67			*
*KRAS*^MUT^/*TP53*^MUT^	19	6	0.663	0.202–2.174	0.562
*KRAS*^MUT^/*TP53*^WT^	21	10			
*KRAS*^WT^/*TP53*^MUT^	112	64	2.321	1.240–4.347	0.009*
*KRAS*^WT^/*TP53*^WT^	65	16			

Note:

* P<0.05 was considered to indicate statistical significance.

Abbreviations: OR, odds ratio; CI, confidence interval; MUT, mutant type; WT, wild-type.

**Table 3:** Multivariate analysis of the treatment response (n = 264).

Variable	OR	95% CI	P
Age (≥65 vs. <65)	1.945	1.089–3.474	0.025*
PD-L1 (positive vs. negative)	2.444	1.354–4.409	0.003*
TMB (high vs. low)	2.706	1.478–4.954	0.001*
Treatment line (1^st^ line vs. 2^nd^ or later)	2.093	1.077–4.065	0.029*
Regimen (ICIs alone vs. ICIs plus chemotherapy)	0.409	0.205–0.814	0.011*

Note:

* P<0.05 was considered to indicate statistical significance.

Abbreviations: OR, odds ratio; CI, confidence interval; PD-L1, programmed death-ligand 1; TMB, tumor mutation burden; ICI, immune checkpoint inhibitor.

**Fig 2:**
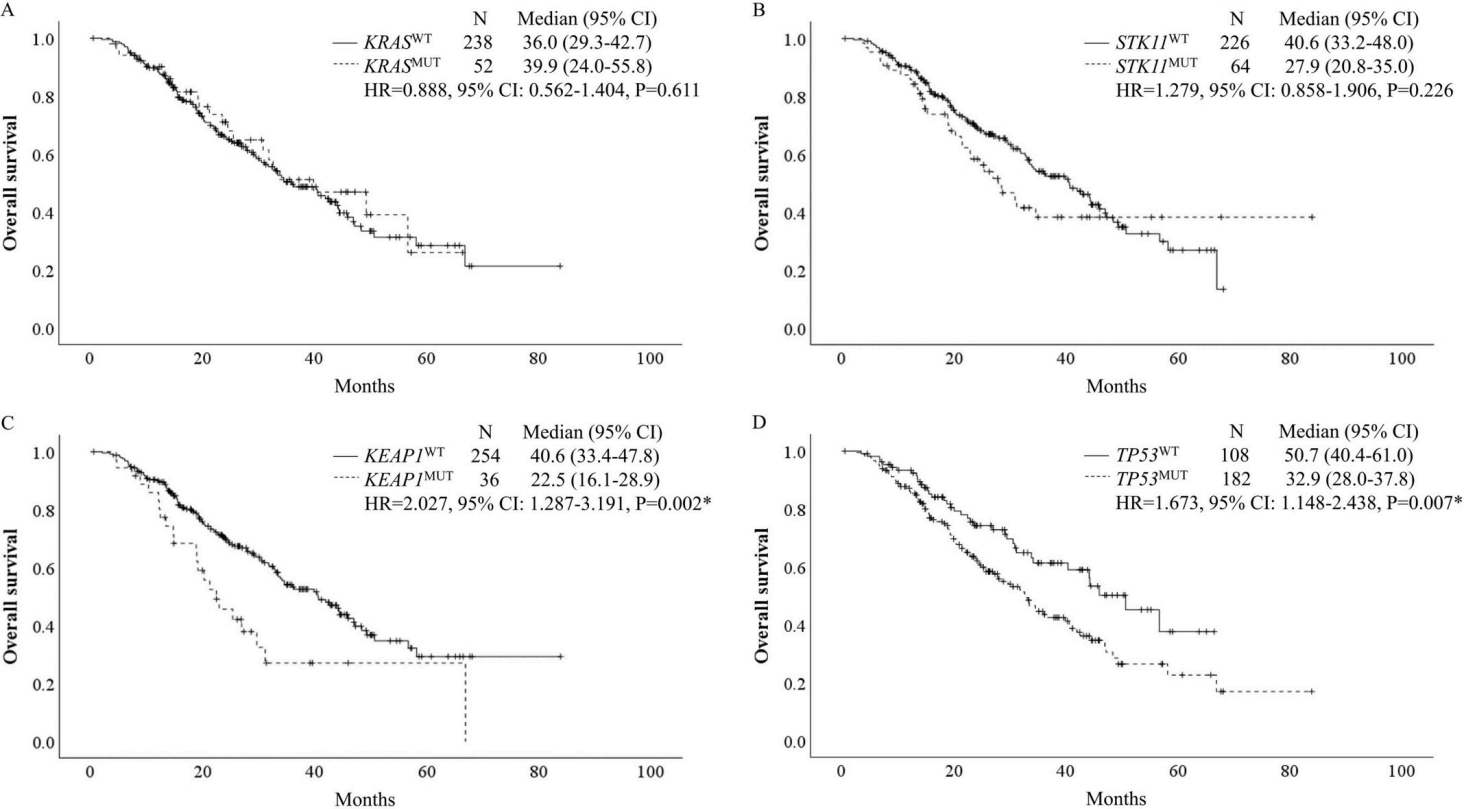
OS according to the single-gene status of *KRAS*, *STK11*, *KEAP1*, and *TP53*. Kaplan–Meier curves of OS in patients with lung adenocarcinoma according to the single-gene status of (A) *KRAS*, (B) *STK11*, (C) *KEAP1* and (D) *TP53*. Note: * P<0.05 was considered to indicate statistical significance. Abbreviations: OS, overall survival; CI, confidence interval; WT, wild-type; MUT, mutant-type; HR, hazard ratio.

**Fig 3:**
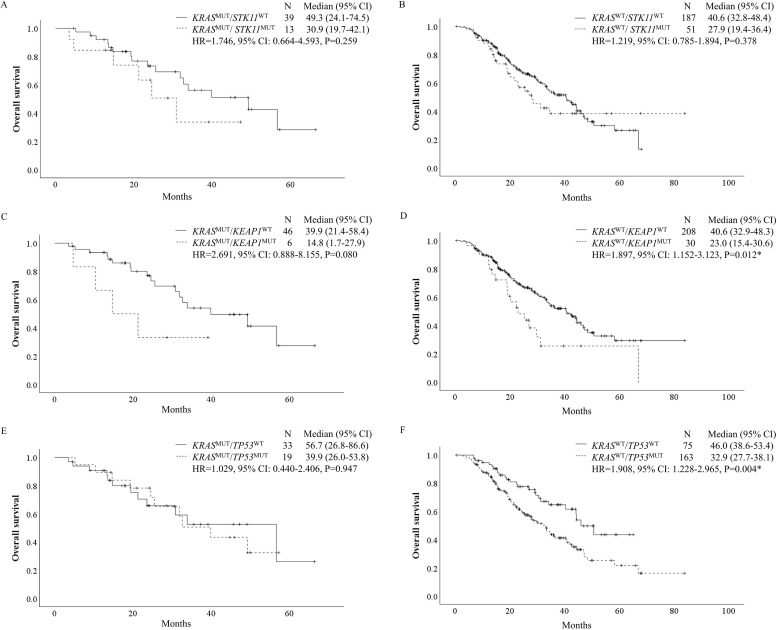
OS according to the comutation status of *KRAS* and *STK11*, *KEAP1* or *TP53*. Kaplan–Meier curves of OS according to the *STK11* status among patients with (A) *KRAS*^MUT^ and (B) *KRAS*^WT^ lung adenocarcinoma. Kaplan–Meier curves of OS according to the *KEAP1* status among patients with (C) *KRAS*^MUT^ and (D) *KRAS*^WT^ lung adenocarcinoma. Kaplan-Meier curves of OS according to the *TP53* status among patients with (E) *KRAS*^MUT^ and (F) *KRAS*^WT^ lung adenocarcinoma. Note: * P<0.05 was considered to indicate statistical significance. Abbreviations: OS, overall survival; CI, confidence interval; WT, wild-type; MUT, mutant-type; HR, hazard ratio.

OS was calculated according to the status of *KRAS*, *STK11*, *KEAP1*, and *TP53* for 290 patients whose survival data were available (Fig 2). No significant difference was observed in OS among patients with or without *KRAS* or *STK11* mutations, whereas OS was significantly shorter in patients with *KEAP1* (HR, 2.027, 95% CI: 1.287–3.191, P = 0.002) or *TP53* (HR, 1.673, 95% CI: 1.148–2.438, P = 0.007) mutations than in those without these mutations. When OS was evaluated according to the *KRAS* status, we observed no association between the *STK11* status and OS, while *KEAP1* (HR, 1.897, 95% CI: 1.152–3.123, P = 0.012) and *TP53* (HR, 1.908, 95% CI: 1.228–2.965, P = 0.004) mutations contributed to significantly a shorter OS among patients with wild-type *KRAS* (Fig 3). Additionally, patients with *KRAS* and *KEAP1* mutations tended to have worse OS (HR, 2.691; 95% CI: 0.888–8.155; P = 0.080). The multivariate analysis of OS revealed that ECOG PS 1 or worse (HR, 1.762, 95% CI: 1.192–2.606, P = 0.005), treatment with ICIs plus chemotherapy (HR, 0.470, 95% CI: 0.316–0.698, P<0.001), mutation of *KEAP1* (HR, 1.890, 95% CI: 1.179–3.031, P = 0.008) or *TP53* (HR, 1.735, 95% CI: 1.135–2.654, P = 0.011), and alterations in any other driver gene (HR, 2.244, 95% CI: 1.444–3.487, P<0.001) were associated with inferior OS (Table 4).

**Table 4:** Multivariate analysis of OS (n = 245).

Variable	HR	95% CI	P
ECOG PS (1 or worse vs. 0)	1.762	1.192–2.606	0.005*
Regimen (ICIs alone vs. ICIs plus chemotherapy)	0.470	0.316–0.698	<0.001*
*KEAP1* (mutant vs. wild-type)	1.890	1.179–3.031	0.008*
*TP53* (mutant vs. wild-type)	1.735	1.135–2.654	0.011*
Alterations in any other driver gene (with vs. without)	2.244	1.444–3.487	<0.001*

Note:

* P<0.05 was considered to indicate statistical significance.

Abbreviations: OS, overall survival; HR, hazard ratio; CI, confidence interval; ECOG, Eastern Cooperative Oncology Group; PS, performance status; ICI, immune checkpoint inhibitor.

A sensitivity analysis was performed to investigate the impacts of *KRAS*, *STK11*, *KEAP1*, and *TP53* mutations on OS among patients treated with first- and second-line anti-PD-(L)1 antibodies separately (S2 Table). A significant association between *KEAP1* mutation and worse OS was observed among patients receiving anti-PD-(L)1 antibodies as the first- (P = 0.050) or second-line (P<0.001) treatment. Patients carrying *TP53* mutations who received first-line anti-PD-(L)1 antibodies tended to have shorter OS (P = 0.070). When analyzing the relationships between gene mutations and OS according to the *KRAS* status, *STK11* and *KRAS* comutant-type (P = 0.019) and *KEAP1* mutant but *KRAS* wild-type (P = 0.024) showed statistically significant differences in the OS of patients treated with second-line therapy. We also evaluated the influences of mutations in the four genes on the treatment response and OS among subgroups treated with or without ICIs in combination with chemotherapy combination (S3 and S4 Tables). Patients with mutant *KEAP1* (P = 0.010) and those with mutant *TP53* but wild-type *KRAS* (P = 0.012) were more likely to achieve a better response to ICIs alone than to ICIs in combination with chemotherapy. We found no association between comutations and the treatment response, regardless of the regimen. Comutation of *KRAS* and *KEAP1* was correlated with inferior OS in patients treated with ICIs alone (P = 0.043), whereas no association existed between comutations and OS in patients treated with ICIs plus chemotherapy. *KEAP1* or *TP53* mutation contributed to worse OS in the general patient population (P = 0.006, P = 0.019, respectively) and in the wild-type *KRAS* population (P = 0.004, P = 0.030, respectively) after treatment with ICIs plus chemotherapy.

Because of the small number of patients for whom the FoundationOne® Liquid CDx was used, univariate analyses were only performed to test the associations between the statuses of the four genes and the TMB, the TMB and the treatment response, and the TMB and OS according to two other cancer genomic medicine test methods (data not shown). No association between *KRAS* mutation and the TMB was observed in patients tested using FoundationOne® CDx, but significant associations between *STK11*, *KEAP1*, and *TP53* mutations and the TMB were identified. We obtained similar results for the OncoGuide™ NCC Oncopanel, except for *TP53*. The treatment response was associated with a high TMB in the FoundationOne^®^ CDx group but not in the OncoGuide™ NCC Oncopanel group. Both methods presented no association between the TMB and OS.

## Discussion

In this study, we retrospectively explored the impact of single-gene mutations in *KRAS*, *STK11*, *KEAP1*, or *TP53*, as well as *STK11*, *KEAP1*, or *TP53* mutations according to the *KRAS* status, on the clinical outcomes of patients with lung adenocarcinoma who received ICIs according to data from the C-CAT database. Overall, *STK11*-, *KEAP1*-, or *TP53*-mutant patients presented a significantly greater TMB. On the other hand, patients with PD-L1-positive and TMB-high lung adenocarcinoma tended to exhibit a superior treatment response, which was consistent with previous studies [19] and supported the reliability of the C-CAT database. The benefit of *TP53* mutation among the general and *KRAS* wild-type patient populations in terms of the TMB translated into a minor improvement in the objective response. *KEAP1* mutation promotes immune evasion and immunotherapy resistance [20]. In our study, although a significant association between the gene status and treatment response of any of the evaluated patients was not observed, *KEAP1* mutation contributed to an inferior OS, which supported the findings of a previous report indicating that *KEAP1* mutation is a prognostic factor for patients with lung adenocarcinoma receiving ICIs. Although the effect of *TP53* on the OS of patients treated with ICIs remains controversial [5, 11, 21], our results indicated a negative prognostic role for *TP53* mutation, which was consistent with the role of *TP53* in immune evasion through the regulation of immune checkpoint expression [22].

In addition, although the difference was not significant, nonnegligible differences in the median OS were observed between the subgroups with and without *STK11* mutations, regardless of the *KRAS* mutation status. Among the *KRAS* mutation subgroups, the Kaplan‒Meier curves of patients with or without *KEAP1* mutations were separated after the early stage, and those with *KEAP1* and *KRAS* comutation tended to have worse OS. The absence of a statistically significant association of the comutation might be due to the small sample size. Our results were consistent with the consensus that *STK11* and *KEAP1* mutations are associated with an immunosuppressive tumor microenvironment [23, 24], but the findings were inconsistent with a retrospective study investigating whether the *KRAS* status could affect the efficacy of PD-(L)1 inhibitors among 1,261 patients with *STK11*- or *KEAP1*-mutant lung adenocarcinoma [25]. These results suggested that both *STK11* and *KEAP1* mutations could worsen the objective response, progression-free survival (PFS), and OS among patients with *KRAS* mutations but not among those without *KRAS* mutations, while we only observed that patients with *KEAP1* mutations but wild-type *KRAS* had a shorter OS. Another retrospective study evaluating the clinical outcomes of first-line pembrolizumab according to the *KRAS* and *TP53* statuses among 696 patients with ≥ 50% PD-L1-positive NSCLC reported that *TP53* mutation increased the response rates, PFS, and OS in a *KRAS*-mutant population but not in a *KRAS*-wild-type population [26]. However, we found that *TP53* mutation improved the response but decreased OS among patients without *KRAS* mutation but not among those with *KRAS* mutation. Compared to those studies, our study showed a much longer OS period for each subgroup, possibly due to the heterogeneity of treatment lines and treatment types.

In contrast to the findings of a previous study in which *STK11* and *KEAP1* mutation carriers exhibited decreased tumor proportion scores (TPSs) for PD-L1 expression [25], we only observed that *STK11*-mutant patients and *STK11*-mutant but *KRAS* wild-type patients might be less likely to exhibit PD-L1 positivity. A possible interpretation might be that the statistical analysis for PD-L1 expression was conducted only using recorded data for PD-L1 positivity or negativity since patients’ TPSs of PD-L1 expression were inaccessible. However, compared to single mutations of *KRAS*, *KRAS* and *TP53*, comutations were more strongly associated with PD-L1 positivity, which was consistent with the findings of a previous study [13]. On the other hand, we reported similar results showing that *STK11*, *KEAP1* and *TP53* mutations were associated with an increased TMB [5, 25]. Generally, an increased TMB is associated with longer survival in cancer patients receiving ICIs [27]. However, our results suggested that despite the TMB and PD-L1 status, *KEAP1* and *TP53* mutations were independently associated with decreased OS. A study identifying the relationship between gene mutations, the TMB, and survival in patients with lung adenocarcinoma reported similar findings [28]. Single-gene mutations and comutations in *KEAP1*, *STK11*, *PBRM1*, and *SMARCA4* in patients were accompanied by an elevated TMB and were associated with shorter OS, possibly due to the induction of an immune-cold microenvironment by mutations in those genes.

According to the sensitivity analysis, *KEAP1* mutation was the sole factor associated with a poor prognosis for patients treated with first- or second-line anti-PD-(L)1 antibodies, whereas *TP53* mutation tended to decrease OS only in patients treated with first-line anti-PD-(L)1 antibodies. Although the differences in the findings of these two genes according to the *KRAS* status among patients receiving first- or second-line treatment should be considered, these results should be interpreted cautiously due to the small sample size of each subtype. Regardless of treatment with ICIs alone or in combination with chemoimmunotherapy, comutation of *KRAS* and *STK11* or *KEAP1* was associated with a worse objective response rate and OS, and *KEAP1* mutation among *KRAS*-wild-type NSCLC patients was correlated with inferior OS after chemoimmunotherapy [25, 29]. In our study, we only observed positive associations between mutations in *KEAP1* or *TP53* but wild-type *KRAS* and the treatment response to ICIs alone. No apparent difference in the response to immunotherapy or chemoimmunotherapy was identified according to the comutation status. Despite the trend of associations of *KEAP1* and *TP53* mutations among the general and *KRAS*-wild populations with worse OS in patients treated with ICIs alone, these mutations had more apparent prognostic effects on patients treated with chemoimmunotherapy. Among the comutation subgroups, only the *KRAS* and *KEAP1* comutation subgroup of patients treated with the ICIs alone showed a similar outcome to that in a previous study [25]. A possible interpretation of the discrepancy between our comutation results and those of previous studies might be the small sample sizes of each comutation subgroup and the inclusion of different treatment lines.

In the present study, *KRAS* mutations were detected in 18% of patients with lung adenocarcinoma, but this rate is only approximately 10% among Japanese patients [30]. We did not observe any association between *KRAS* mutation and the clinical response to ICIs. Indeed, findings on the correlation between *KRAS* mutation and ICI efficacy in NSCLC patients are inconsistent [31, 32], making it challenging to draw a definitive conclusion.

To our knowledge, the present study is the first to investigate the impacts of *KRAS*, *STK11*, *KEAP1*, and *TP53* mutations on the clinical outcomes of Japanese patients with lung adenocarcinoma treated with ICIs using the C-CAT database. Our findings revealed associations between mutations in specific genes and the TMB and clinical outcomes of patients with lung adenocarcinoma treated with ICIs, which demonstrated the usefulness of the C-CAT database and may be meaningful in personalized medicine. However, some limitations in this retrospective study should be noted. First, the sample sizes of populations with comutations were small, especially in the *KRAS* and *KEAP1* comutation population. Second, although we compared results from different cancer genomic medicine tests that showed similar results, the results were not completely consistent. Possible interpretations might be the small sample size of the group analyzed using the OncoGuide™ NCC Oncopanel and bias due to genomic testing methods. Third, this study included individuals with alterations in other driver genes in addition to *KRAS* mutations, which could affect the contributions of the four target genes to the outcomes. Fourth, although we performed additional analyses of treatment lines and types, the main analyses included a heterogeneous population, and since we did not analyze subsequent treatment after ICIs, which could affect OS, the conclusion was not sufficiently strong. Fifth, we did not perform a PFS analysis for all patients since data on disease progression were not recorded in the C-CAT database.

In conclusion, *STK11*, *KEAP1*, and *TP53* mutations are significantly associated with a high TMB. *TP53* mutation could affect the treatment response to some degree, and both *KEAP1* and *TP53* mutations resulted in inferior OS in the general patient population and in those with *KRAS-*wild-type lung adenocarcinoma, indicating that *KEAP1* and *TP53* mutations might act as prognostic factors for ICI treatment in lung adenocarcinoma patients.

## Supporting information

S1 Table Univariate analysis of the PD-L1 expression status according to the *KRAS*, *TP53*, *STK11*, and *KEAP1* statuses. Abbreviations: PD-L1, programmed death-ligand 1; OR, odds ratio; CI, confidence interval; MUT, mutant-type; WT, wild-type.
(DOCX)

S2 Table Univariate analysis of OS according to the *KRAS*, *STK11*, *KEAP1*, and *TP53* statuses in patients treated with first- and second-line anti-PD-(L)1 antibodies. Abbreviations: OS, overall survival; PD-(L)1, programmed death-(ligand) 1; HR, hazard ratio; CI, confidence interval.
(DOCX)

S3 Table Univariate analysis of the treatment response according to the *KRAS*, *STK11*, *KEAP1*, and *TP53* statuses in patients treated with ICIs in combination with/without chemotherapy. Abbreviations: ICI, immune checkpoint inhibitor; OR, odds ratio; CI, confidence interval.
(DOCX)

S4 Table Univariate analysis of OS according to the *KRAS*, *STK11*, *KEAP1*, and *TP53* statuses in patients treated with ICIs in combination with/without chemotherapy. Abbreviations: OS, overall survival; ICI, immune checkpoint inhibitor; HR, hazard ratio; CI, confidence interval.
(DOCX)
