# Correction: Effects of *KRAS, STK11, KEAP1*, and *TP53* mutations on the clinical outcomes of immune checkpoint inhibitors among patients with lung adenocarcinoma

**DOI:** 10.1371/journal.pone.0330099

**Published:** 2025-08-11

**Authors:** Yao Liang, Osamu Maeda, Chiaki Kondo, Kazuki Nishida, Yuichi Ando

There are errors in the author affiliations. The correct affiliations are as follows:

Yao Liang^1^, Osamu Maeda^2^, Chiaki Kondo^2^, Kazuki Nishida^3^, Yuichi Ando^2^

**1** Division of Clinical Oncology and Chemotherapy, Department of Integrated Medicine, Nagoya University Graduate School of Medicine, Nagoya, Aichi, Japan, **2** Department of Clinical Oncology and Chemotherapy, Nagoya University Hospital, Nagoya, Aichi, Japan, **3** Department of Advanced Medicine, Nagoya University Hospital, Nagoya, Aichi, Japan
