# Supplementary material for: Effects of KRAS, STK11, KEAP1, and TP53 mutations on the clinical outcomes of immune checkpoint inhibitors among patients with lung adenocarcinoma
Source: PLoS One. 2024 Jul 22;19(7):e0307580. doi: 10.1371/journal.pone.0307580 (PMC11262633; doi:10.1371/journal.pone.0307580)
Supplement: S1 Table — Abbreviations: PD-L1, programmed death-ligand 1; OR, odds ratio; CI, confidence interval; MUT, mutant-type; WT, wild-type. (DOCX) [file pone.0307580.s001.docx]

S1 Table. Univariate analysis of the PD-L1 expression status according to the *KRAS*, *STK11*, *KEAP1*, and *TP53* statuses.

| Gene status | No. of patients without PD-L1 positivity | No. of patients with PD-L1 positivity | OR | 95% CI | P |
| --- | --- | --- | --- | --- | --- |
| *KRAS*^MUT^ | 22 | 34 | 1.398 | 0.773-2.530 | 0.299 |
| *KRAS*^WT^ | 114 | 126 |  |  |  |
| *TP53*^MUT^ | 82 | 113 | 1.583 | 0.976-2.568 | 0.066 |
| *TP53*^WT^ | 54 | 47 |  |  |  |
| *STK11*^MUT^ | 34 | 29 | 0.664 | 0.380-1.161 | 0.157 |
| *STK11*^WT^ | 102 | 131 |  |  |  |
| *KEAP1*^MUT^ | 17 | 22 | 1.116 | 0.566-2.200 | 0.863 |
| *KEAP1*^WT^ | 119 | 138 |  |  |  |
| *KRAS*^MUT^/*STK11*^MUT^ | 6 | 8 | 0.821 | 0.240-2.802 | 0.762 |
| *KRAS*^MUT^/*STK11*^WT^ | 16 | 26 |  |  |  |
| *KRAS*^WT^/*STK11*^MUT^ | 28 | 21 | 0.614 | 0.326-1.157 | 0.150 |
| *KRAS*^WT^/*STK11*^WT^ | 86 | 105 |  |  |  |
| *KRAS*^MUT^/*KEAP1*^MUT^ | 2 | 4 | 1.333 | 0.223-7.980 | 1.000 |
| *KRAS*^MUT^/*KEAP1*^WT^ | 20 | 30 |  |  |  |
| *KRAS*^WT^/*KEAP1*^MUT^ | 15 | 18 | 1.100 | 0.526-2.300 | 0.853 |
| *KRAS*^WT^/*KEAP1*^WT^ | 99 | 108 |  |  |  |
| *KRAS*^MUT^/*TP53*^MUT^ | 5 | 20 | 4.857 | 1.450-16.266 | 0.013* |
| *KRAS*^MUT^/*TP53*^WT^ | 17 | 14 |  |  |  |
| *KRAS*^WT^/*TP53*^MUT^ | 77 | 93 | 1.354 | 0.775-2.366 | 0.321 |
| *KRAS*^WT^/*TP53*^WT^ | 37 | 33 |  |  |  |
| Note: * P<0.05 was considered to indicate statistical significance.  Abbreviations: PD-L1, programmed death-ligand 1; OR, odds ratio; CI, confidence interval; MUT, mutant-type; WT, wild-type. | | | | | |
