# Supplementary material for: Effects of KRAS, STK11, KEAP1, and TP53 mutations on the clinical outcomes of immune checkpoint inhibitors among patients with lung adenocarcinoma
Source: PLoS One. 2024 Jul 22;19(7):e0307580. doi: 10.1371/journal.pone.0307580 (PMC11262633; doi:10.1371/journal.pone.0307580)
Supplement: S2 Table — Abbreviations: OS, overall survival; PD-(L)1, programmed death-(ligand) 1; HR, hazard ratio; CI, confidence interval. (DOCX) [file pone.0307580.s002.docx]

S2 Table. Univariate analysis of OS according to the *KRAS*, *STK11*, *KEAP1*, and *TP53* statuses in patients treated with first- or second-line anti-PD-(L)1 antibodies.

| Variable | First line | | | Second line | | |
| --- | --- | --- | --- | --- | --- | --- |
|  | HR | 95% CI | P | HR | 95% CI | P |
| *KRAS* (mutant vs. wild-type) | 0.566 | 0.255-1.257 | 0.162 | 1.413 | 0.596-3.350 | 0.432 |
| *STK11* (mutant vs. wild-type) | 1.708 | 0.936-3.117 | 0.081 | 1.717 | 0.768-3.843 | 0.188 |
| *KEAP1* (mutant vs. wild-type) | 1.924 | 1.001-3.698 | 0.050* | 8.036 | 2.619-24.652 | <0.001* |
| *TP53* (mutant vs. wild-type) | 1.824 | 0.953-3.492 | 0.070 | 0.982 | 0.478-2.019 | 0.961 |
| *KRAS* mutant-type + *STK11* (mutant vs. wild-type) | 1.282 | 0.233-7.058 | 0.775 | 15.574 | 1.560-155.492 | 0.019* |
| *KRAS* mutant-type + *KEAP1* (mutant vs. wild-type) | 2.968 | 0.540-16.296 | 0.211 | 5.634 | 0.777-40.835 | 0.087 |
| *KRAS* mutant-type + *TP53* (mutant vs. wild-type) | 1.572 | 0.351-7.046 | 0.554 | 0.427 | 0.080-2.293 | 0.321 |
| *KRAS* wild-type + *STK11* (mutant vs. wild-type) | 1.755 | 0.923-3.338 | 0.086 | 1.127 | 0.421-3.013 | 0.812 |
| *KRAS* wild-type + *KEAP1* (mutant vs. wild-type) | 1.698 | 0.835-3.452 | 0.144 | 5.553 | 1.247-24.735 | 0.024* |
| *KRAS* wild-type + *TP53* (mutant vs. wild-type) | 1.798 | 0.856-3.775 | 0.121 | 1.410 | 0.587-3.390 | 0.442 |
| Note: * P<0.05 was considered to indicate statistical significance.  Abbreviations: OS, overall survival; PD-(L)1, programmed death-(ligand) 1; HR, hazard ratio; CI, confidence interval. | | | | | | |
