# Supplementary material for: Effects of KRAS, STK11, KEAP1, and TP53 mutations on the clinical outcomes of immune checkpoint inhibitors among patients with lung adenocarcinoma
Source: PLoS One. 2024 Jul 22;19(7):e0307580. doi: 10.1371/journal.pone.0307580 (PMC11262633; doi:10.1371/journal.pone.0307580)
Supplement: S3 Table — Abbreviations: ICI, immune checkpoint inhibitor; OR, odds ratio; CI, confidence interval. (DOCX) [file pone.0307580.s003.docx]

S3 Table. Univariate analysis of the treatment response according to the *KRAS*, *STK11*, *KEAP1*, and *TP53* statuses in patients treated with ICIs in combination with/without chemotherapy.

| Variable | ICIs alone | | | ICIs plus chemotherapy | | |
| --- | --- | --- | --- | --- | --- | --- |
|  | OR | 95% CI | P | OR | 95% CI | P |
| *KRAS* (mutant vs. wild-type) | 1.059 | 0.440-2.550 | 1.000 | 0.773 | 0.296-2.019 | 0.638 |
| *STK11* (mutant vs. wild-type) | 1.758 | 0.731-4.227 | 0.225 | 0.809 | 0.344-1.903 | 0.671 |
| *KEAP1* (mutant vs. wild-type) | 2.996 | 1.293-6.938 | 0.010* | 1.191 | 0.428-3.319 | 0.796 |
| *TP53* (mutant vs. wild-type) | 1.784 | 0.674-4.723 | 0.274 | 1.093 | 0.520-2.299 | 0.852 |
| *KRAS* mutant-type + *STK11* (mutant vs. wild-type) | 0.733 | 0.591-0.910 | 0.315 | 1.111 | 0.142-8.680 | 1.000 |
| *KRAS* mutant-type + *KEAP1* (mutant vs. wild-type) | 1.143 | 0.102-12.784 | 1.000 | 1.714 | 0.092-31.924 | 1.000 |
| *KRAS* mutant-type + *TP53* (mutant vs. wild-type) | 2.083 | 0.412-10.529 | 0.443 | 0.122 | 0.012-1.300 | 0.085 |
| *KRAS* wild-type + *STK11* (mutant vs. wild-type) | 2.575 | 1.008-6.580 | 0.058 | 0.761 | 0.297-1.953 | 0.640 |
| *KRAS* wild-type + *KEAP1* (mutant vs. wild-type) | 1.963 | 0.675-5.707 | 0.229 | 1.116 | 0.373-3.337 | 1.000 |
| *KRAS* wild-type + *TP53* (mutant vs. wild-type) | 3.578 | 1.289-9.931 | 0.012* | 1.555 | 0.652-3.705 | 0.388 |
| Note: * P<0.05 was considered to indicate statistical significance.  Abbreviations: ICI, immune checkpoint inhibitor; OR, odds ratio; CI, confidence interval. | | | | | | |
