# Supplementary material for: Effects of KRAS, STK11, KEAP1, and TP53 mutations on the clinical outcomes of immune checkpoint inhibitors among patients with lung adenocarcinoma
Source: PLoS One. 2024 Jul 22;19(7):e0307580. doi: 10.1371/journal.pone.0307580 (PMC11262633; doi:10.1371/journal.pone.0307580)
Supplement: S4 Table — Abbreviations: OS, overall survival; ICI, immune checkpoint inhibitor; HR, hazard ratio; CI, confidence interval. (DOCX) [file pone.0307580.s004.docx]

S4 Table. Univariate analysis of OS according to the *KRAS, STK11, KEAP1,* and *TP53* statuses in patients treated with ICIs in combination with/without chemotherapy.

| Variable | ICIs alone | | | ICIs plus chemotherapy | | |
| --- | --- | --- | --- | --- | --- | --- |
|  | HR | 95% CI | P | HR | 95% CI | P |
| *KRAS* (mutant vs. wild-type) | 1.001 | 0.568-1.765 | 0.997 | 0.780 | 0.353-1.722 | 0.539 |
| *STK11* (mutant vs. wild-type) | 1.023 | 0.571-1.834 | 0.938 | 1.422 | 0.824-2.523 | 0.199 |
| *KEAP1* (mutant vs. wild-type) | 1.797 | 0.943-3.427 | 0.075 | 2.528 | 1.308-4.886 | 0.006* |
| *TP53* (mutant vs. wild-type) | 1.415 | 0.875-2.289 | 0.157 | 2.112 | 1.133-3.939 | 0.019* |
| *KRAS* mutant-type + *STK11* (mutant vs. wild-type) | 2.144 | 0.653-7.034 | 0.209 | 1.115 | 0.181-6.853 | 0.906 |
| *KRAS* mutant-type + *KEAP1* (mutant vs. wild-type) | 3.916 | 1.045-14.672 | 0.043* | 1.085 | 0.120-9.829 | 0.942 |
| *KRAS* mutant-type + *TP53* (mutant vs. wild-type) | 0.837 | 0.293-2.386 | 0.739 | 1.246 | 0.273-5.680 | 0.776 |
| *KRAS* wild-type + *STK11* (mutant vs. wild-type) | 0.844 | 0.427-1.670 | 0.626 | 1.553 | 0.856-2.816 | 0.147 |
| *KRAS* wild-type + *KEAP1* (mutant vs. wild-type) | 1.459 | 0.689-3.086 | 0.324 | 2.828 | 1.406-5.688 | 0.004* |
| *KRAS* wild-type + *TP53* (mutant vs. wild-type) | 1.719 | 0.968-3.053 | 0.064 | 2.176 | 1.080-4.383 | 0.030* |
| Note: * P<0.05 was considered to indicate statistical significance.  Abbreviations: OS, overall survival; ICI, immune checkpoint inhibitor; HR, hazard ratio; CI, confidence interval. | | | | | | |
